# Analysing change in music therapy interactions of children with communication difficulties

**DOI:** 10.1098/rstb.2015.0374

**Published:** 2016-05-05

**Authors:** Neta Spiro, Tommi Himberg

**Affiliations:** 1 Research Department, Nordoff Robbins, 2 Lissenden Gardens, London NW5 1PQ, UK; 2 Department of Neuroscience and Biomedical Engineering, Aalto University, Rakentajanaukio 2C, 02150 Espoo, Finland

**Keywords:** music therapy, interaction, communication, video analysis

## Abstract

Music therapy has been found to improve communicative behaviours and joint attention in children with autism, but it is unclear what in the music therapy sessions drives those changes. We developed an annotation protocol and tools to accumulate large datasets of music therapy, for analysis of interaction dynamics. Analysis of video recordings of improvisational music therapy sessions focused on simple, unambiguous individual and shared behaviours: movement and facing behaviours, rhythmic activity and musical structures and the relationships between them. To test the feasibility of the protocol, early and late sessions of five client–therapist pairs were annotated and analysed to track changes in behaviours. To assess the reliability and validity of the protocol, inter-rater reliability of the annotation tiers was calculated, and the therapists provided feedback about the relevance of the analyses and results. This small-scale study suggests that there are both similarities and differences in the profiles of client–therapist sessions. For example, all therapists faced the clients most of the time, while the clients did not face back so often. Conversely, only two pairs had an increase in regular pulse from early to late sessions. More broadly, similarity across pairs at a general level is complemented by variation in the details. This perhaps goes some way to reconciling client- and context-specificity on one hand and generalizability on the other. Behavioural characteristics seem to influence each other. For instance, shared rhythmic pulse alternated with mutual facing and the occurrence of shared pulse was found to relate to the musical structure. These observations point towards a framework for looking at change in music therapy that focuses on networks of variables or broader categories. The results suggest that even when starting with simple behaviours, we can trace aspects of interaction and change in music therapy, which are seen as relevant by therapists.

## 1. Introduction

In improvisational music therapy the client and therapist improvise music together. The sessions are happening for therapeutic purposes, but music therapy is seen by many people as part of the spectrum of music making that we all have. In other words, the aspects we would usually associate with music making, including aesthetic beauty or interpersonal communication or interaction are considered integral to the process [1–4]. Although music therapy has been found to improve communicative behaviours and joint attention for some clients [5], there is a lack of clarity regarding what in the music therapy sessions drives those changes. In order to explore characteristics related to changes in communicative behaviour and joint attention, we investigate the interaction between the client and therapist. Our aim is to identify features of joint behaviours that would be relevant to the aims of the therapy sessions, while also being comparable across different sessions, therapists and clients. We map simple, relatively unambiguous behaviours that can be coded from video recordings: movement and facing behaviours, rhythmic activity and musical structures. We focus on how these behaviours are shared in the pair (their mutuality). We track differences and similarities between clients, therapists, client–therapist pairs and pairs in different sessions early or late in the therapeutic process. Our intention is not to develop new outcome measures for assessing music therapy, but rather provide tools that would help to understand the process of music therapy better, and in particular, the interaction between the therapist and the client.

Here we focus on music therapy with clients who are rarely able to speak in detail about their experiences of the sessions and for whom one of the main reasons they are in music therapy is to aid aspects of interaction and communication. Indeed, they all have difficulties with speech and most have a diagnosis of autism spectrum disorder (ASD). As discussed below, the specific characteristics that are featured in the discussion of music and music therapy research are highly relevant for autism and children with communication difficulties. We therefore discuss research about capacities necessary for interaction and communication in autism and in music as well as exploring the importance of considering both participants in a collaborative activity. There is a long-standing history of the use of video analysis in music therapy research and the analysis of change in music therapy, and we explore some examples of such approaches before presenting the current approach.

## 2. Interaction in music therapy sessions and communication difficulties

Some children who come to music therapy have communication difficulties, and some of them have diagnoses of ASDs. ASD is described as including ‘[p]ersistent deficits in social communication and social interaction across multiple contexts' and ‘[r]estricted, repetitive patterns of behaviour, interests, or activities' [6]. What exactly the consequences are for these characteristics in music therapy should be further explored, particularly given the range of mechanisms and processes relevant to autism that may be relevant in music therapy [7]. There has been a lot of attention on music therapy with children with a diagnosis of autism [8,9] and it is not our purpose here to test whether music therapy has particular ‘effects' or to directly connect between ‘outcomes' and process.

Children with communication difficulties and ASD often have difficulties with many aspects of joint actions from an early age. For example, difficulties with attention, motor tonus, initiative and emotion, prospective control of movements and anticipations in awareness have been related to asynchronous social behaviour. This, in turn, frustrates carers' attempts to support activities such as learning to walk, share toys or play games. This has been related to further confusion on the parts of carers, and a further reduction in mutual attention and joint activity [10]. These timing-related deficits can adversely affect turn-taking in communication. Turn-taking is a very time-sensitive form of mutual entrainment. Music therapy can provide support for learning interpersonal timing, and rewarding experiences of successful turn-taking [11–13]. More generally, a deficit in joint attention has been a well-documented feature of autism [14–16] where there seems to be reduced neural responses to faces [17] or social scenes [18]. For example, children with autism seem to look at social stimuli for less time than their typically developing counterparts.

Motor activity, motor coordination, motor imitation, postural stability as well as timing may be problematic for children with autism [19–21]. The exact relationship between social difficulties and motoric ones is still debated [19], but this relationship is often played out in music therapy. For example, music therapy is often considered helpful for children with autism precisely because it sets up a predictable frame for action in time and therapists adjust their actions to those of the children [3]. Also, improvement in eye contact—with consequences interpreted for joint attention—has been found in music therapy studies with this client group [8].

In music, as in other communicative activities such as conversation, the importance of facing each other for the interaction to be successful seems to vary depending on the nature of the activity [22,23]. Indeed, even if there are differences in some aspects of communication when visual channels are open or closed, this may not necessarily affect the content of the interaction [24]. In music, different situations require different levels of visual interaction. For example, in one study synchronization was seen to be better for highly skilled performers when they could see each other compared with when they could not [25]. Conversely, another study suggested that accompanists do not necessarily synchronize more accurately when they can see their partners [26]. Indeed, there are many musical creations that proceed well when musicians cannot see each other, such as in some recording studios or in duo improvisation with a visual barrier between the players [27]. For children playing together, the contribution of being able to see each other may be positive or negative [28]. More specifically, whether or not musicians keep visual track of each other (mutually or unidirectionally) may depend on a number of factors including physical constraints, how well they know each other, how much they have played music or a particular piece together and whether what they are playing is predictable or not. When we face our collaborators in music making, we can keep track of them visually at different levels from the most to least peripheral, or not at all. Taken together, these observations mean that while it is important to quantify the amount of time the client and therapist are facing each other, individually or mutually, we do not start from specific hypotheses about whether facing each other more is better or not.

Music can continue without players continuously facing each other, as musical information and in particular pulse, provides the necessary predictability regarding when to play next. Indeed, commonly cited reasons for the success of music therapy include the matching of individuals' ‘basic beat’ or pulse in a musical framework [2,5] and the predictability of a pulse structure allowing continued music making. The constant adjustments involved in entrainment, or at least unidirectional synchronization, allow players to stay together [29].

Seeing each other and having a shared pulse are two possible routes to making music. In some cases of musical interaction, both may be needed; for example, when the pulse is agreed but visual contact is needed to monitor or control changes in musical roles such as accompanying or playing solo. In other cases, the two may be interchangeable. For example, looking at each other is no longer needed when a regular pulse is established, and needed again only to stop, or after a longer pause to coordinate restarting. Sharing one's own attentional resources between one's own part and listening to the other person's part is a relatively complex cognitive task [30]. On one hand, the attentional demands are higher than in everyday conversations and other social encounters, but on the other hand, especially rhythmic structure and predictability, as well as the flexibility of musical meaning, serve as scaffolding that can foster the development of attention sharing skills [31].

Other factors are likely to affect how much players face each other or play together. One basic factor may be whether they are still or travelling around the room. It is often easier to face someone and to play in time if players are still rather than walking around. Being still may also indicate concentration over distractedness. Conversely, moving around the room to music and moving around the room together are both joint activities.

## 3. It takes two

Much analysis in which understanding of music and musical processes was developed was dominated by focus on one person (e.g. [32]). However, in recent years, it has become apparent that the kinds of processes that dominate in dyadic or group interactions are quite different from the solo situation (e.g. [33]). For instance, in considering pulse capacities, focus on one person can lead to prioritizing pulse accuracy. Focus can be on the individual's ability to adjust to the target pulse as well as the characteristics of that adjustment [32]. Scaling the task up to more than one person raises questions about mutual adaptation and about what the ‘target’ now is. It also raises the possibility of a co-created musical product, as well as directing the attention of the researcher towards the interaction of the dyad [29,34].

Such observations are not limited to music. For example, rather than seeing conversation—and language use more generally—simply as the sum of a speaker speaking and a listener listening, it should be seen as a joint action. This joint action emerges when speakers and listeners—writers and readers—perform their individual actions in coordination, as ensembles [35].

Similarly, improvisational music therapy includes both the therapist and the client. Some analyses—especially analyses of change—focus entirely on clients [8]. Others acknowledge therapists to some extent in analysis of sessions and understanding of clients [2]. Interaction involves both therapists and clients and the therapists do not follow an identical unresponsive protocol of behaviour in sessions and, therefore, cannot be treated as a ‘controlled’ variable. Rather their contribution has been observed to be ‘significant for the therapeutic musical relationship’ [36, p. 28]. The roles and levels of expertise in music therapy are multifaceted. For example, on one hand, the therapist is treating all interactions as musical and aiming to produce an aesthetic product in which the client's sounds are seen as musical and equally important to the final product. On the other hand, the relationship in dyadic interaction in music therapy, especially with children with autism, is not necessarily symmetrical. The therapist knows more about music making and its conventions and goals, and has different motoric and cognitive capacities from the client. Similarly, assumptions about mutuality and synchrony that one could make in professional music making are not so readily available in this context. In any case, we see it as essential, when looking at change in interaction, to consider both therapist and client.

## 4. Assessment and video analysis in MT

Assessment tools form part of a wide network of processes by which some music therapists trace their clients' progress [37,38]. Some assessment scales in music therapy use video material to assess change. Although we do not propose an assessment system, our analysis tool does focus on tracing changes in interaction using video material. Therefore, here we briefly compare our system with examples of existing assessment tools. There are many characteristics that could be included in assessment and in analysis of videos of sessions, requiring more or less interpretation on the part of the annotator [39–41]. Current assessment tools have some limitations, which we tried to avoid in developing our analysis tools. For example, most assessment systems are focused mostly on the client, and are not designed for comparisons between therapists or clients. Furthermore, current assessment systems are complex and require subjective interpretation of the client's behaviour, or even their intentions. It is also common for assessment tools to require a lot of knowledge about the context of the therapy session, such as the diagnosis of the client, or what took place in previous sessions.

A recent scale, for example, has the purpose of assessing ‘musical-play interactions' with people with neurodevelopmental disorders [41]. Its first subscale examines musical–social–emotional capacities in musical-play such as the ‘ability to attend, respond affectively, adapt, engage, and interrelate’. Using video segments from sessions, the therapist rates the frequency of each target response, the support provided by the therapist, and whether it was instrumental, vocal or a movement. These ratings require subjective judgement by the therapist according to rather broad categories, which are, in turn, based on a number of more detailed judgements. Some of these are summarized in the text of the paper that uses the scale. For example, ‘Jason displays islands[…] of capacity in the area of musical attention during relational music making[…] While he exhibited the ability to focus in musical-play independently, he presented with constrictions in maintaining, sharing, and shifting attention when the music intensified rhythmically and dynamically. This generally lead him to become dysregulated in an over-reactive manner, thus withdrawing from the musical interaction and engaging in perseverative movements and play. When dysregulated, however, Jason could easily be redirected back into musical-play with verbal and visual cuing’ [41, p. 57]. These observations too are based on more detailed judgements that remain implicit in the scale. It is, therefore, difficult to trace all the steps from observation to judgement of assessment for those not involved in the sessions. This assessment method stays close to the experience of the therapist and relies on familiarity with the client. While the richness of description above is out of the reach of our simpler annotation method, our method does not require prior knowledge of the context. This means that people other than the original therapist can carry out the analysis. Moreover, sessions from multiple therapists can be included in the analysis. Also, the reliability of the annotations can be systematically assessed, something that is relatively difficult in the case of current assessment systems.

Beyond use in assessment of music therapy, videos are among the main ways in which information about music therapy is shared both in practice and research. In case studies, moments for analysis are often carefully chosen for a specific reason, usually by the therapist(s) who participated in the sessions (e.g. [2]). In large-scale research projects, moments are chosen according to rules that are separated from the activities in the videos, such as always choosing the same time-span in the session (e.g. [8]). These are perhaps the only feasible ways to identify video material for analysis. For both, it is difficult to understand context of moments for those not involved in music therapy process. It is difficult to know if, or in what way, observations are generalizable (or assumed to be generalizable) and what aspects of the work are being used as the basis for generalization. One motivation for sticking to simple behaviours in our system is to make it possible to annotate and analyse larger corpora of music therapy videos, so that we can also see what happens in music therapy outside these highlight reels.

There are many studies that are close to practice in which the music therapists narrate and analyse what happened in the sessions. This contributes to the reflective and reflexive nature of this practice. However, people's reports of their experiences of music making can be rather different (and indeed people can change their minds about what they thought) [27] and so taking the music therapist's view as the only one perhaps misses other aspects of what may be going on. This is particularly important in the context of work with clients who may not be able to talk about their interpretations of what happened in sessions.

Both assessment tools and case studies can require interpretations of intentions of the client, assigning behaviours and acts with meanings that might not be accurate or appropriate. Such complexities when the studier and studied overlap have been recognized in psychology research—our human capacities to be affected by what we know and what we want to see are certainly not specific to music therapy (for an example concerning the use of confederates in language studies see [42]). In some cases, these complexities are secondary to the main aim of the work and so can be acknowledged and even celebrated: for some questions the person best placed to describe what happened is the expert. However, for our purposes, we propose a different and complementary method of exploration. We start with behaviours that are simple and observable in the videos also by outsiders who have no background information about the client or what happened in previous sessions.

## 5. Change in interaction in music therapy

Music therapy often works towards change and, in many cases, change in interaction. Practising interaction skills in the musical context is, among other things, seen as a way to strengthen social skills generally. However, representing and exploring what changes, and how, is still debated. One perspective prioritizes the idea that each client and each session is different and that therapists, therefore, adapt and respond to each client in each session differently. In this line of thinking, change can occur in different domains (musical or social) and changes in individual characteristics can occur in different directions, depending on the client. Indeed, it is possible for some clients to be very strict and predictable in their generation of pulse; in such cases therapists might work towards helping clients become more flexible and responsive. Other clients may arrive playing a rather erratic pulse and the therapist might work towards playing more consistently. In such a view, case studies are particularly relevant forms of enquiry which detail exactly what changes and how, and there are many examples of this in the music therapy literature (e.g. [43]).

From another perspective, some characteristics are expected to change in a particular way and direction. In such cases, randomized controlled studies seem particularly relevant (such as [8]). Indeed, in recent years, there has been an increase in the number of studies that assess outcome of music therapy using specific—and pre-specified—measures. This necessitates the identification of a relatively small number of characteristics (such as initiating or responding to eye contact, responses to pointing gestures and turn-taking [5]). These assess the client and often implicitly take the therapist's actions as a given.

On the basis of previous research, such as that discussed so far on music, music therapy, music psychology and autism, and theories about communication, interaction and mutuality, one could draw up a series of hypotheses about what is expected to be challenging for these clients, what therapists are likely to be working on, and what change might look like. Put simply, characteristics associated with joint action could be expected to be ‘better’ when comparing early and later sessions. However, given the wide range of individual differences in clients, the variability of methods employed by the therapists, and the multitude of ways in which music therapy sessions might progress, in this study we do not generate specific hypotheses but rather chart a set of behaviours relevant for joint attention and musical interaction. Many of these individual features might interact with each other, and they might serve different communicative purposes in different musical and social contexts.

Of course, it is likely that there are some levels of interpretation and aspects of change in interaction that are common among clients and others that are more client-specific. For example, assessing change at a level of interpretation that focuses on general responsiveness in the music is rather different from assessing change in how many times a client responds to a pointing gesture by the therapist. One challenge then, is to identify a level that is helpful in understanding change and is feasible and reliable in terms of analysis.

In exploring change in this context, we face basic questions: How can we represent at least some aspects of what happens in music therapy sessions? How can we observe whether there is change in aspects to do with interaction? Which aspects should we be observing? The goal of this project is to develop an approach that allows for comparison among many therapy sessions, between different clients and therapists, to explore at what level patterns are generalizable. In this paper, we illustrate this approach in a relatively small sample of client–therapist interactions (10 sessions), and ask: what are the similarities and differences between these music therapy sessions, between players, pairs and the same pairs' different sessions?

## 6. Material and methods

### (a) Participants

All music therapists working in the Nordoff Robbins London Centre were invited to submit videos from their archives that fulfilled the following inclusion and exclusion criteria: (i) one-to-one work with clients, aged 4–7 years, with a diagnosis of autism, referred for help with communication (most clients were also referred for other reasons); (ii) videos from the first and final three sessions. The ‘final’ session had to have been a part of an intentional ending. Clients may have since returned; (iii) sufficient video quality for analysis: complete sessions recorded both in audio and video; client and therapist in view for the majority of the time. Therapists suggested their work with eight clients. One client was excluded as it was no longer appropriate to contact their family for consent, but the rest consented. Two client–therapist pairs were excluded because the client–therapist pair could not be seen for large parts of the sessions.

We remained with five client–therapist pairs. All five therapists were Nordoff Robbins-trained music therapists. Of the five clients, four were male and they all were aged 4–5 years at the start of music therapy. They all had communication difficulties among the reasons for referral or therapists' diagnoses. All had a diagnosis of ASD except MK who had a non-specified communication disorder. This variety of clients and session structures (see §6b) reflect rather well the situation in Nordoff Robbins London Centre, where therapists see a wide variety of clients, many of whom may not have a specific diagnosis when they arrive. Rather than trying to select a sample that is as uniform as possible (as would be advisable for an outcome study), we included a group that reflects a common range of clients with communication difficulties, as our aim is to develop tools that could be applied as broadly as possible.

Through a questionnaire, the five music therapists who participated in the study were asked to rate the extent to which each of the annotated features was relevant to describing the development of their clients in music therapy. They were presented with moment-by-moment visualizations of the annotations with basic descriptions of them and asked how easy they were to understand, how useful they could be, and whether they had learned from them.

### (b) Videos

**Figure 1.**
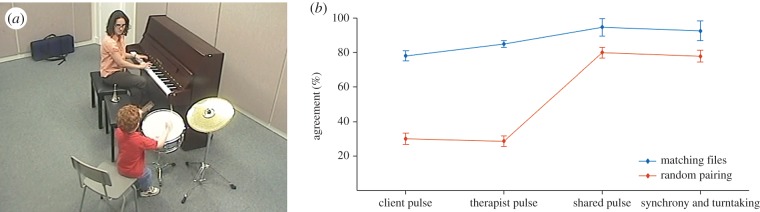
(*a*) Screenshot from the video data. (*b*) Agreement between rater and co-rater (blue) in four pulse-related tiers, compared with a baseline of unrelated pairings (red). Error bars represent s.e.m.

For each client–therapist pair, we analysed two videos of one-to-one sessions, one early in the therapeutic process (one of the first three sessions for the pair) and the other one late (one of the last three sessions). The sessions were on average (±s.d.) 11 (±7.7) months apart, and on average 27.8 (±3.6) min in duration. They were recorded in one or two cameras installed in the top corners of the room (see figure 1*a* for a screenshot). The audio is recorded using room microphones.

### (c) Annotation protocol

There are many characteristics that could be analysed and several studies have focused on a range of characteristics that require more or less interpretation on the part of the annotator (e.g. [39–41]). We start from the simplest possible types of annotation (table 1). Our criteria for characteristics are that they should be: (i) clearly observable and should not require interpretation of intention, mood or emotional state; (ii) considered as important in social interaction (and simultaneously, by definition, in music making); (iii) should afford the analysis of social interaction in a musical context. (The detailed annotation protocol is in the electronic supplementary material.) The analyses presented in this paper are based on annotations concerning four aspects: (1) where the clients and therapists are facing individually (electronic supplementary material, table S1) and relative to each other (electronic supplementary material, table S2); (2) whether they stay in one place or move around the room individually (electronic supplementary material, table S1) and relative to each other (electronic supplementary material, table S2); (3) their individual pulse characteristics (electronic supplementary material, table S3) and the relationship between them (electronic supplementary material, table S4); (4) characteristics of the structure of the music played (electronic supplementary material, table S5). In the analysis below, these aspects are referred to, respectively, as (1) facing, (2) still, (3) pulse and (4) musical structure.

**Table 1.** Features and annotation options. For details of the definitions for each annotation value, see the electronic supplementary material.

	annotation options	
feature	individual	mutual
facing	facing	both facing each other
	not facing	both not facing each other
	out of view	one facing the other and one not
still	still	both still
	not still	both not still
	out of view	one still and one not
pulse	regular	shared pulse
	irregular	not shared pulse
	non-pulsed musical sounds	
	non-musical sounds	
	silence	
musical structure		song
		free improvisation

### (d) Tools

Video recordings were annotated using ELAN software by the Max Planck Institute for Psycholinguistics, Nijmegen [44]. The annotations were processed and analysed in MATLAB, using custom-made ELAN-MATLAB functions [45], built on the SALEM Toolbox [46].

## 7. Results

### (a) Reliability of annotation protocol

Coding of the videos was carried out by experts with experience in both music therapy and scientific research. To evaluate the reliability of the annotation protocol, 10% of each video (3 min) was coded by a second coder. The re-coding was done to four tiers related to client's and therapist's pulse (with annotation options for regular, irregular, non-pulsed musical sounds, non-musical sounds, silence), shared pulse (with options yes and no) and synchrony (with annotation options synchrony and turn-taking), as these were considered the most complicated ones. Sections for re-coding were chosen in all the videos in which both the client and therapist were active during the given time window. The re-coded section was the same in all cases: the section between 5 and 8 min in the session.

Agreement between the first and second coders was assessed by calculating the percentage of time points during which the two had the same annotation. The timeline of annotations was sampled at 0.1 s resolution, leading to an average of 1800 samples per timeline to be compared. As the percentage of agreement is likely to yield higher results the fewer annotations there are in the tier, the results were compared with agreement percentages obtained from a matching sample of surrogate data, created by pairing annotations from two unrelated videos. The observed agreement for the actual pairs and baselines obtained from the surrogate data for the four tiers we looked at are in figure 1*b*.

The two first tiers, documenting the client's and music therapist's pulse characteristics, had a lot of annotations, on average 50 in the 3-min section. The shared pulse and synchrony, and turn-taking tiers were much more sparse, with only 11 annotations on average (see also figure 4). Therefore, the higher agreement in the latter two tiers was expected. Importantly, *t*-tests confirmed that inter-rater agreement was higher than the baseline in all four occasions: 95% CIs ranged from 44.7–67.5 for the MT pulse to 5.6–23.7 for the synchrony tier.

### (b) Individual and mutual

Here we present two ways of visualizing the data. The first provides an overview for the sessions, while the second looks at moment-by-moment representations of sessions.

#### (i) Therapists

**Figure 2.**
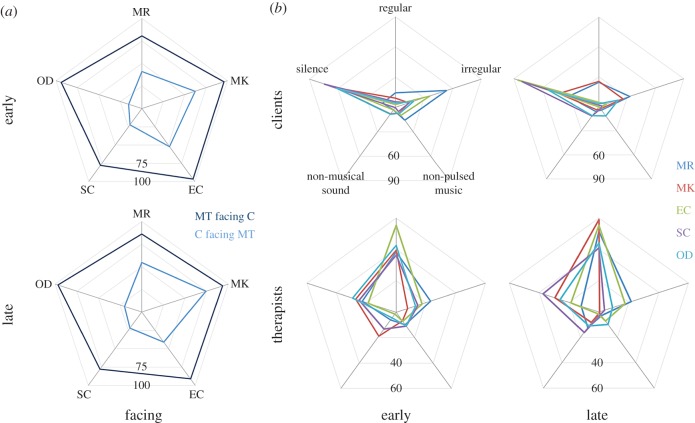
(*a*) All therapists face the clients almost all the time (dark blue), and change very little. Clients face therapists less (light blue), but this increases by the late session in some pairs. (*b*) Individual pulse behaviours plotted separately for clients and therapists, early and late sessions.

Some characteristics were rather consistent across therapists. For example, all were facing the client most of the time (figure 2*a*).^1^ Other characteristics, such as pulse, were consistent in the first session but less so in the later session (figure 2*b*). One interpretation of this change is that these therapists shared a common approach to how to begin a music therapy process. In music therapy training, the provision of a frame and a clear musical structure is emphasized [2,3]. This can be done with a combination of cues including rhythmic structure. In these sessions, this did not translate to playing music every second of the session, but we can observe that there was an emphasis on regular pulse. For the later session (figure 2*b*, bottom right), the differences between the therapists may be interpreted as being related to their adaptation towards the clients; the difference may reflect their responses to what they see as the individual client's needs or their music making.

#### (ii) Clients

These patterns of consistency and difference contrast with variability in at least some of these characteristics for the clients. The amount of time clients spent facing the therapist differed both between clients and between sessions (figure 2*a*), and the time facing the therapist could change in either direction. Similarly, the proportion of time clients spent playing a regular pulse (figure 2*b*) varied dramatically (2.8–14% in the early session and 2.9–25% in the later session) with changes possible in both directions. Clients differed in how much sound they made and within that the types of pulse they produced in both sessions (figure 2*b*). We observed both increase (+16.3%^2^ and +10.1%), decrease (–4%) and little change (+1% and –1%), for example, in the proportion of regular pulse between sessions.

#### (iii) Client and therapist profiles

**Figure 3.**
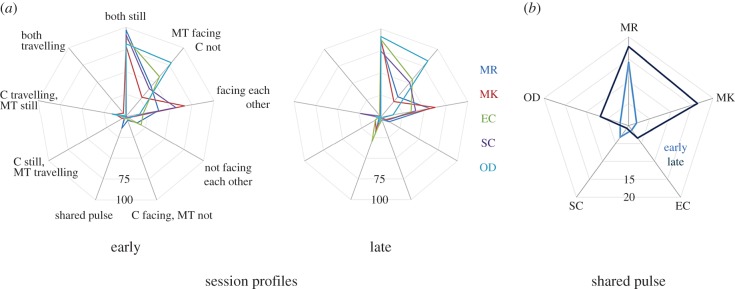
(*a*) Session profiles show the interrelationships of facing, being still and pulse. (*b*) Shared pulse across all pairs, comparison of early (light blue) and late (dark blue) sessions.

At a general level, certain behaviours were much more dominant than others (stillness, facing each other and client not facing the therapist while the therapist faces the client, figure 3*a*). However, each profile differs from the others in the details both in the early sessions and the direction of change. Clearly, these individual behaviours are not independent. In the later session, for example, the client in the SC pair is less still (16.7% less of the time); it is not surprising that there is less mutual facing too (approx. 16.5% less). The proportion of shared pulse out of sound produced is low—in all cases, clients and therapists spend more time making sounds not in a shared pulse than in a shared pulse (figure 3*b*). At a more detailed level, the shared pulse characteristics of each pair can be different (figure 2*a*). Generally, there is relatively little shared pulse (1.2–14.3% in the early session and 0.7–17.8% in the later session). As before, we see that change can go both ways: with both an increase and decrease in shared pulse as observed in this way.

In very broad patterns then, there are similarities between the pair profiles, but some aspects are different. It will be crucial to explore what types or levels of difference are significant for what kinds of client–therapist development and outcome. Now that we have a way of reaching these representations it will be possible to go back to other sources (other annotations, reasons for referrals, characterizations by the therapists and others) to see how these observations relate (or not).

### (c) In the session: case example

**Figure 4.**
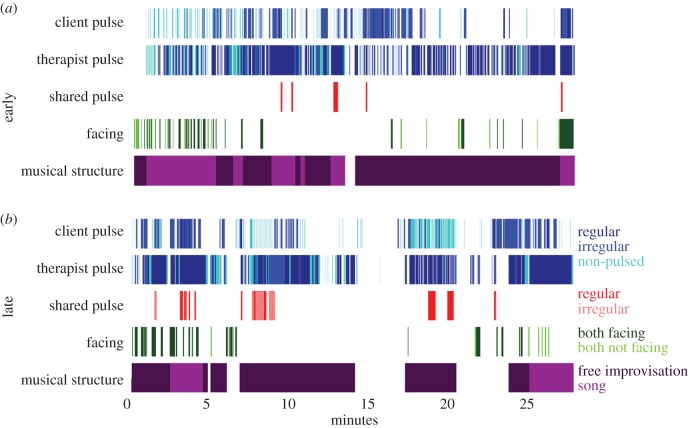
A timeline visualization of one pair's (OD) early (*a*) and late (*b*) sessions. (Note: in some parts of these sessions, the therapist or client is out of view. In both of these sessions this is mainly in the middle of the sessions. This is of particular relevance for the mutual facing tier.)

The client–therapist profiles above have the advantage of describing, at a glance, information about several pairs simultaneously. However, the temporal structure of the sessions also needs to be unfolded, so that we can see how the relationship between the client and the therapist develops during the session, and what the temporal relationship is between different characteristics. This can be done by visualizing the annotations of selected tiers as a timeline (figure 4).

In terms of individual pulse, both players switch between different pulse categories throughout the session and in some moments they both coincided in a shared pulse. Both client and therapist had more and longer bouts of regular and shared pulse in the later session than the earlier one. Most of the client's regular bouts coincided with the therapist's regular pulse but not vice versa: often the therapist was playing regularly and the client was not.

In terms of mutual facing, some segments of the session seem to be dominated by the client and therapist facing each other for brief moments while others have periods when they are not facing each other. Looking at shared pulse in the context of mutual facing, it seems that they mostly did not overlap: this pair either faced each other or shared a pulse (or neither).

Both sessions include sections of structured music (songs) and others that are free improvisation. The structured music consists either of structural songs (‘hello’ and ‘goodbye’ songs) that book-end the session, or structured and/or known songs (such as nursery rhymes) some of which are constructed to encourage structurally relevant responses from the client. The first session was more dominated by these songs than the later one. Within this context, we see that the shared pulse bouts occurred almost exclusively during songs in the first session, while in the later session they occurred during both songs and improvised music.

### (d) Therapists' views

Preliminary analysis of the responses of three of five of the therapists suggests that all features discussed in this paper were relevant to describing their clients' developments in music therapy and that the images and associated descriptions were easy to understand. They could see these representations being useful for their own analyses of sessions, especially maintaining a balance between being able to focus on details and having an overview of the whole session.

Two therapists found that the images revealed something surprising to them. One was not aware of how much structured song there had been in the early session (OD). The other (EC) commented that, ‘[h]aving seen the sessions, I perceived an increase in facing each other in the late session. However, I might have been ‘seeing’ this togetherness through the increase in a shared play.’ This remark might hint that different characteristics can be seen to serve similar functions in interaction.

Of course, the images do not immediately represent all of the therapists' observations. For example, one therapist commented that their client was ‘able to tolerate interaction and engagement and changed from being distressed and resistive to being relaxed and enjoying interaction, musical and non musical’ (EC). The images for this client illustrate the longer sustained periods of playing, and the more time the client and therapist spend facing each other. However, the visualizations do not provide the interpretation of why these changes occur. In another example, a therapist observes that ‘in the earlier session I am predominantly following my client, he responds by noticing and laughing when I match his music but I am always following his lead. In the later session he is much more likely to respond to me and sustain his musical interaction with me’ (OD). Again, the therapist provides a richer description of what happens in the session, but aspects of these observations can be seen in the images, with more occurrences of shared pulse in the later than in the earlier session.

## 8. Conclusion and future work

Session videos of five client–therapist pairs were annotated according to a protocol that aimed to map simple and unambiguous characteristics of each pair's behaviour—their individual and mutual facing, moving in the room, pulse and musical structure. The lack of ambiguity of the annotations was supported by the high inter-rater reliability, and even though the behaviours themselves are quite simple, the combinations of them within the pair (mutuality of the behaviours), as well as interconnections between them, turned out to be informative.

The global views of the clients and therapists, the profiles of interaction and the detailed views of sessions can be seen as complementary: each has its possibilities and limitations and switching from one to the other in the analysis helps to put each in context.

Analysis of the individual characteristics raised some striking results, including that shared pulse occupies a small proportion of the sessions. The criteria in place for identification of shared pulse are relatively stringent: sounds need to be synchronized or within a clear rhythmic structure. Moreover, in the analysis presented here, we treat pulse as occurring only in the auditory domain. However, it is possible to create pulsed movements and then share pulse in movement only and across music and movement. In our examples, the SC pair has a segment in which the therapist plays the piano and sings and the client runs and jumps around the room often in synchrony with the music. This is not captured in the current annotation. Indeed, the criteria for the pulse annotations used here arose from the view that we prioritize the musical sounds in improvisational music therapy and that analysis can be seen through relatively fine-grained musical and music-theoretic categories. However, we could have used other views of mutuality, or at least togetherness or responsiveness to music, that might have been be more general. For example, one can imagine investigating relative frequency of movement or sound rather than specific pulse [47].

The profiles of interaction can be, at a broad level, quite similar across pairs, but there is variation in the details. This perhaps goes some way to reconciling the two views of client- and context-specificity on one hand and generalizability on the other. Not surprisingly, whether the characteristics of behaviour are seen as common or idiosyncratic may be related to the level of analysis. For example, broadly speaking we see that mutual facing is dominant and that the alternative is the therapist facing the client and not vice versa. However, the exact proportions of each of these, and how much they change and in what direction, may be part of a web of factors (see [27, p. 4] for discussion of a recognition of individual differences alongside generalizability).

The changes in the inter-therapist consistency highlight two aspects of this music therapy work. First that it is a mutual process. It seems that there is a general principle of beginning by providing a frame—interpreted at the level of pulse in rather similar ways across therapists—followed by learning from the client. But the therapist is not a consistent model to be imitated and does not follow a standard direction of change.

Second, any change that is seen in the client is dependent on the relationship with the therapist: just as the client is in a process of change during their therapy, the therapist is too. This is typical of most social relationships. (In conversation, for example, both parties mutually develop their understanding and use techniques such as repair [48].) Nevertheless, it raises questions about whose judgement we take when assessing ‘client’ progress, and indeed what can be judged as ‘client’ progress if looking at the client–therapist pair.

The moment-by-moment visualization example indicated some differences between early and late sessions, with both players having more and longer bouts of regular and shared pulse in the later session. Often the therapist was playing regularly and the client was not. The mutual facing and the shared pulse tiers mostly did not overlap: this pair either faced each other or shared a pulse (or neither). In this case it seems that these two modes are two alternative ways of being together.

The first session was more dominated by songs than the later one. This may again highlight an aspect of this music therapy work: early on in a series of sessions perhaps there is more focus on provision of explicit structure through songs, with later sessions having music that is more freely improvised. Within this context of change from more structured to more free improvisation, we see in the case example that the shared pulse bouts occurred almost exclusively during songs in the first session, while in the later session they occurred in both songs and in improvised music.

These visualizations of sessions allow us to start identifying whether these and other such patterns occur across different client–therapist pairs, and whether our examples are representative of larger groups or outliers. We can then explore whether they are related to other factors, for example, clients' diagnoses. To fully explore this, it would of course be necessary to see if these findings replicate in a larger sample of client–therapist pairs, or clients with other diagnoses. Larger samples would allow us to conduct statistical analyses, such as looking at durations of the bouts of behaviours and how they are distributed in time. It would also allow us to analyse how behaviours are related to one another, on one hand in terms of coincidence and on the other in terms of complementarity.

Coding these tiers is very time consuming, and such analysis is not feasible for huge datasets. This paper may help in the selection of shorter segments for more detailed analysis depending on the goal of the research. Alternatively, data could be collected using technologies such as motion and touch sensors and MIDI instruments. Such tools may also help in the exploration of aspects of mutuality that were not possible in the current study. For example, we can only identify synchrony between players (note onsets that sound like they begin at the same moment), not entrainment (mutually adapting note-onset times in response to each other [29,32,34,49]). Clearly, many other characteristics would be interesting, such as physical proximity or different types of turn-taking (e.g. [8,13]).

We saw in the case example that mutual facing and shared pulse tend not to co-occur. It may be that other pairs will show a different relationship (and more overlap between cues). Either way, such observations help with the exploration of what we take as representative of communication and what we take as representing change. If we take mutual facing as the only variable, we would see little change. If we take shared pulse, we see more. At some level, perhaps, these may be seen as different instantiations of types of communication and could be treated as such. This possibly points towards a different framework for looking at change in music therapy: rather than looking at individual variables as representative, looking at networks of variables or at broader categories could be more appropriate.

The views of those who participated in these sessions are important. Preliminary findings suggest that the features explored in this method are relevant to therapists' analyses of these sessions, and the resulting visualizations are useful in their consideration of change in the interaction with their clients. Given that the therapists see these representations as being relevant to practice, such representations may help make predictions about what we expect to change within and beyond the therapy room.

Although improvisational music therapy sessions serve therapeutic purposes, the music created in them is a part of the wide spectrum of music making, and thus these methods and results could be relevant for studies of emotion, interpersonal interaction and body movement, especially in the context of music. Interest in multimodal aspects of communication is growing, and indeed some efforts towards an integrative analysis of multiple social signals have been made, with promising results (see e.g. [50–52]), and the links between musical and verbal domains have recently been explored [53,54]. For example, both hardware and software for multimodal study of musical interaction have been developed in the SIEMPRE project.^3^ However, these set-ups tend to require complicated laboratory facilities and/or manipulation of participants that are not feasible in this music therapy context. Our study could be relevant not only to other music therapy researchers but to the field of social interaction in general. The software and data analysis methods are openly available. The basic principle of developing an analysis of social interaction using annotations of basic behaviours can be applied to almost any study of social interaction. By contrast, the annotation protocol itself needs to be tailored to the video data and the context.

The annotation protocol and methods of data analysis in this project develop an integrative approach to analysing multimodal data (musical, physical and gestural)—an approach that seems essential in an attempt to capture the complexity of this real-world social interaction.

## Supplementary Material

Annotation Protocol

## Endnotes

1 During some parts of the session, the therapist and/or client are out of view. In these cases, it is not possible to annotate where they are facing or whether they are still or moving. For these characteristics, in all overview figures, 100% refers to the total time both client and therapist are in view.

2 For MK.

3 For SIEMPRE Project Consortium, see http://www.infomus.org/siempre/.

## Data accessibility

The annotation data used in this paper, as well as the MATLAB functions used in analysing and visualizing the annotation files, are available online at http://tijh.github.io/ELAN-MATLAB.
