# Supplementary material for: Analysing change in music therapy interactions of children with communication difficulties
Source: Philos Trans R Soc Lond B Biol Sci. 2016 May 5;371(1693):20150374. doi: 10.1098/rstb.2015.0374 (PMC4843612; doi:10.1098/rstb.2015.0374)
Supplement: Annotation Protocol [file rstb20150374supp1.pdf]

Supplementary material for Spiro and Himberg, 2016, Analysing change in music therapy interactions of children with communication difficulties, *Phil. Trans. R. Soc. B.* doi: 10.1098/rstb.2015.0374

Annotation protocol

There are many characteristics that could be analysed. Here we present four aspects of the annotation protocol. These include information about:

- where the clients are facing individually (table 1) and relative to each other (table 2).
- whether they stay in one place or moving around the room individually (table 1) and relative to each other (table 2),
- their individual pulse characteristics (table 3) and their relationship (table 4).
- characteristics of the structure of the music played (table 5).

**Annotation**	**Rule**
Facing	Player is facing the other player
Not facing	Player is not facing the other player
Still	Player does not take more than two steps in one direction
Not Still	Player takes more than two steps in one direction[1]

Supplementary Table 1: Where players are facing and whether they are still or moving

On the basis of these annotations, we can then automatically combine annotations, using the function "Create annotations from overlaps" in ELAN, to reach the following annotations.

**Characteristic**	**Annotation**
Facing	Both facing each other
	Both not facing each other
	Client is facing therapist and therapist is not facing client
	Therapist is facing client and client is not facing therapist
Still	Both still
	Both not still
	Client is still and therapist is not
	Therapist is still client and client is not

Supplementary Table 2 Characteristics regarding where players are facing and whether they are still of moving that are automatically generated on the basis of original annotations

The pulse characteristics are again annotated in two stages. First, the characteristics of each player are annotated separately. We aim here to account for every moment in the session, therefore we also include annotations for non-pulsed sounds, non musical sounds and silence.

A pulse consists of a series of musical articulations. These are produced directly from an instrument or voice (not including speech or body movement separate from an instrument). To be coded as a **pulse** (whether regular or not), these articulations are to have a clear rhythmic profile: there must be three or more onsets, with less than a two-second gap between the most frequent.	
Regular Pulse	The articulations can be accommodated within a regular metric framework
Irregular Pulse	The articulations cannot be accommodated within a regular metric framework

[1] In the case of standing from a seated position (followed by walking at least two steps), a person starts travelling as soon as they begin to stand. In the case of a person sitting down after travelling, they are considered to have stopped once they are fully in the seated position.

Non-pulsed musical sounds	Musical sounds that do not have the above rhythmic profile. These must be produced directly on an instrument, or with the voice (excluding speech).
Non-musical sounds	All other sounds, including speech and moving (rather than directly sounding) instruments
Silence	The rest of the session but does include sounds such as coughing or heavy breathing.

Supplementary Table 3 Individual players' pulse

Having annotated the pulse characteristics of each player, the characteristic of shared pulse is annotated.

Shared Pulse	Both the music therapist and client have the same pulse, regardless of leadership. They may play synchronously or take turns within the same pulse structure. Where shared pulse is changing (slowing down together, getting faster together), the period between onsets is technically irregular, but they are shared between client and therapist.
Not Shared Pulse	Both the music therapist and client play during the same time but not in the same pulse and not synchronously.

Supplementary Table 4 Relationship between players' pulse

Music therapy sessions can include a wide range of different types of music. Here we annotate broad categories that describe function and structure of musical material (e.g. Hello Songs are usually played at the start of sessions, are usually similar week-to-week and their structure is usually clear). The music-type is annotated and the broad musical categories of Song and Free Improvisation are used in the analysis. The annotation process takes the music at any moment and so makes a global judgement regarding what is being played whether by therapist, client or both

**Characteristics of structure**	**Music type**	**Description**	**Indicators include**
**Song**	Hello Song	Standard repeated tune week-to-week.<br>Clear structure.	c A verbal greeting is used in the lyrics (hello, good morning etc.)<br>c Begins at the start of a session<br>c Usually the same melody week-to-week
	Goodbye Song	As an extended part of final activity or an NR standard song.<br>Clear structure.	c The word goodbye is used in the lyrics.<br>c Begins near the end of a session<br>c Usually the same melody week-to-week
	Nursery Rhyme	MT plays/ sings a known nursery rhyme.<br>Clear structure.	c A familiar nursery rhyme is played / sung by the MT
	Instrumental Song	Client plays instrument while therapist facilitates this playing or	c Instrument(s) is/are used by the client<br>c MT holds instrument to client OR MT accompanies client's playing on another instrument (could be vocal)<br>c Structurally, an instrumental response is

		accompanies on another instrument. There is a pre-ordained structure –not completely free-flow.	required from C for the song to 'work' e.g. the therapist calls for a beat at the end of a phrase or turn taking.<br>c Lyrics of song may instruct or comment upon the client's actions.<br>c May be improvisatory in nature, but theme recurs week to week.
	Vocal Song	As above except client primarily vocalizes	c As above except client vocalises and lyrics of song may instruct or comment upon the client's actions.
Free Improvisation	Free Improvisatio n	Completely free improvisation.	c Therapist and/or client play any instrument / vocalise<br>c No pre-prepared musical material

Supplementary Table 5: Structure of music played

© The Authors under the terms of the Creative Commons Attribution License http://creativecommons.org/licenses/by/3.0/, which permits unrestricted use, provided the original author and source are credited.
